# Targeted sequencing analysis pipeline for species identification of human pathogenic fungi using long-read nanopore sequencing

**DOI:** 10.1186/s43008-023-00125-6

**Published:** 2023-09-06

**Authors:** Nattapong Langsiri, Navaporn Worasilchai, Laszlo Irinyi, Piroon Jenjaroenpun, Thidathip Wongsurawat, Janet Jennifer Luangsa-ard, Wieland Meyer, Ariya Chindamporn

**Affiliations:** 1 Medical Microbiology, Interdisciplinary Program, Graduated School, Chulalongkorn University, Bangkok, Thailand; 2 Department of Transfusion Medicine and Clinical Microbiology, Faculty of Allied Health Science, Chulalongkorn University, Bangkok, Thailand; 3 Immunomodulation of Natural Products Research Group, Chulalongkorn University, Bangkok, Thailand; 4 Westmead Clinical School, Sydney Medical School, Faculty of Medicine and Health, The University of Sydney, Sydney, NSW Australia; 5 Sydney Institute for Infectious Diseases, The University of Sydney, Sydney, NSW Australia; 6 Curtin Medical School, Curtin University, Perth, Bentley, WA Australia; 7 Department of Biomedical Informatics, College of Medicine, University of Arkansas for Medical Sciences, Little Rock, AR USA; 8 Division of Medical Bioinformatics, Faculty of Medicine, Siriraj Hospital, Mahidol University, Bangkok, Thailand; 9 National Center for Genetic Engineering and Biotechnology (BIOTEC), National Science and Technology Development Agency (NSTDA), 111 Thailand Science Park, Phahonyothin Road, Klong Nueng, Klong Luang, Pathum Thani Thailand; 10 Westerdijk Fungal Biodiversity Institute, KNAW, Utrecht, The Netherlands; 11 Department of Microbiology, Faculty of Medicine, Chulalongkorn University, Bangkok, Thailand

**Keywords:** Internal transcribed spacer (ITS), Targeted long-read sequencing, Nanopore technology, Fungal identification

## Abstract

Among molecular-based techniques for fungal identification, Sanger sequencing of the primary universal fungal DNA barcode, the internal transcribed spacer (ITS) region (ITS1, 5.8S, ITS2), is commonly used in clinical routine laboratories due to its simplicity, universality, efficacy, and affordability for fungal species identification. However, Sanger sequencing fails to identify mixed ITS sequences in the case of mixed infections. To overcome this limitation, different high-throughput sequencing technologies have been explored. The nanopore-based technology is now one of the most promising long-read sequencing technologies on the market as it has the potential to sequence the full-length ITS region in a single read. In this study, we established a workflow for species identification using the sequences of the entire ITS region generated by nanopore sequencing of both pure yeast isolates and mocked mixed species reads generated with different scenarios. The species used in this study included *Candida albicans* (n = 2), *Candida tropicalis* (n = 1), *Nakaseomyces glabratus* (formerly *Candida glabrata*) (n = 1), *Trichosporon asahii* (n = 2), *Pichia kudriavzevii* (formerly *Candida krusei*) (n = 1), and *Cryptococcus neoformans* (n = 1). Comparing various methods to generate the consensus sequence for fungal species identification, the results from this study indicate that read clustering using a modified version of the NanoCLUST pipeline is more sensitive than Canu or VSEARCH, as it classified species accurately with a lower abundance cluster of reads (3% abundance compared to 10% with VSEARCH). The modified NanoCLUST also reduced the number of classified clusters compared to VSEARCH, making the subsequent BLAST+ analysis faster. Subsampling of the datasets, which reduces the size of the datasets by approximately tenfold, did not significantly affect the identification results in terms of the identified species name, percent identity, query coverage, percentage of reads in the classified cluster, and the number of clusters. The ability of the method to distinguish mixed species within sub-populations of large datasets has the potential to aid computer analysis by reducing the required processing power. The herein presented new sequence analysis pipeline will facilitate better interpretation of fungal sequence data for species identification.

**Supplementary Information:**

The online version contains supplementary material available at 10.1186/s43008-023-00125-6.

## INTRODUCTION

Fungal infections have a significant increase in prevalence, particularly in tropical regions such as Thailand (Chayakulkeeree and Denning 2017). Traditionally, morphological approaches that use macroscopic and microscopic characteristics have been employed to identify fungi. However, these approaches have major limitations, including the need for specialized personnel, difficulty in identifying morphologically similar species, and the slow growth rate of fungal cells, leading to delayed species identification and disease diagnosis. Molecular approaches, specifically targeted sequencing using polymerase chain reaction (PCR) to amplify species-specific DNA regions or DNA barcodes, have been proposed as a more effective identification method.

In clinical mycology, the internal transcribed spacer (ITS) of the ribosomal region of the nuclear genome has been selected as the primary fungal DNA barcode for species identification (Irinyi et al. 2015; Schoch et al. 2012). The ITS region contains non-coding regions which are flanked by the ribosomal small subunit (SSU) or 18S subunit sequences at the 5' end and the large subunit (LSU) or 28S subunit sequences at the 3' end. The ITS region includes two parts, ITS1 and ITS2, which are separated by the 5.8S subunit (Lafontaine and Tollervey 2001). The main advantage of the ITS region is the ease of amplification by universal primers that bind to conserved regions at the end of the 18S and beginning of the 28S region, enabling PCR amplification and Sanger sequencing from small clinical samples. Furthermore, PCR amplification success rates are typically high (Stielow et al. 2015), and the length of the ITS region is relatively short, making it suitable for Sanger sequencing. An additional major advantage of the ITS region is the availability of many high-quality reference sequences deposited in various online databases (Irinyi et al. 2015; Nilsson et al. 2019; Pruitt et al. 2005; Ratnasingham and Hebert 2007; Schoch et al. 2014).

PCR-based identification and sequencing of the ITS region have been widely used in clinical mycology due to the urgent need for timely diagnosis of fungal infections, which is a significant challenge. However, intragenomic variation within individuals, especially in fungi, has been reported in several studies, increasing intraspecies variation (Colabella et al. 2018; Paloi et al. 2022). Intraspecies variation is a concept in molecular genetics used to measure the degree of polymorphism within the sequence of individuals in a population. This variation has important implications for the ITS region's suitability as a species identification tool, as polymorphisms and heterogeneity within an individual's genome in the same population could increase the distance among individuals within the same species and narrow the distance between different species (Irinyi et al. 2015). Although expanding the region for identification beyond the ITS to include the intergenic spacer region (IGS) (Morrison et al. 2020) or even the entire ribosomal gene cluster (D’Andreano et al. 2021) has been proposed, the variation problem has not yet been addressed, despite the benefits of advanced sequencing technologies, such as third-generation sequencing. Furthermore, the addition of more variable regions, such as the IGS exacerbates the problem, as the number of reference sequences deposited in databases is limited, with fewer full ribosomal gene cluster sequences being publicly available compared to just the ITS sequences.

Among the various sequencing technologies available, Sanger sequencing, which is commonly used in routine analysis, can easily overlook variations among individuals within the population of the same species, as it calls the nucleic acid bases from the consensus signal (Alanagreh et al. 2017). However, in the scenario of mixed infection, this principle can sabotage the consensus signal and aberrate the identification results. This limitation has led to the use of next-generation sequencing (NGS). Early NGS technologies, usually collectively called second-generation sequencing, are characterized by high-throughput short-read sequencing. By inserting an adapter sequence, the sequence can bind with the flow cell and initiate the sequencing cycle, which creates millions of reads in a short time. However, this technology produces very short sequences, which are even shorter than the ones generated by Sanger sequencing. A pipeline for generating the consensus sequence used for identification and inspecting variation within an individual strain using short-read sequencing technology has been developed (Colabella et al. 2018). Although its application showed an efficient way to sequence and inspect the variation that dealt with the inflated species identification caused by the intraspecies variation using NGS technology, it still poses limitations. These limitations include the sequencing technology itself and the bioinformatic analysis that restricts the usage of a pipeline, including the computational cost, limitation of de novo-based identification, and the complexity of the bioinformatic pipeline in contrast with the throughput of the technology. All these factors delayed the establishment of short-read NGS-based species identification in the routine clinical laboratory.

Recently, long-read sequencing has been explored to overcome the limitations of previous technologies and use the benefits of high-throughput sequencing to address the problem of overlooking individual variation (Edgar 2018; Mafune et al. 2019), benefiting its application in epidemiology or evolutionary studies and facilitate identification in the case of mixed infection. One of the most well-known technologies of long-read sequencing nowadays is the nanopore sequencing technology. The principle of nanopore sequencing is that the DNA passes through a nanometer pore channel protein (Jain et al. 2016). A significant advantage of this technology is that the actual sequencer, called MinION™, is much smaller in size, has a more user-friendly interface, and requires less installation and maintenance cost, making it suitable for use in less specialized laboratories or even as point-of-care testing in the future (Jain et al. 2016). Using these features enables the sequencing of all DNA present in an amplicon as a basis to determine all the ITS sequences amplified from a clinical sample.

NGS generates high read numbers for each of the sequences obtained. Traditionally, the sequences are grouped based on their homologies into operational taxonomic units (OTUs). In microbiome analysis, those OTUs represent the taxa/species present, and the number of reads represents the abundance of those species. For short reads that have a higher accuracy per base compared with nanopore’s read the clustering steps could simply be done by relying on the sequence similarity using the greedy clustering algorithm. However, such an algorithm depends on the accuracy of the sequences when used with the noisy reads of nanopore’s sequences, which could result in generating the OTU with the incorrect representative, leading to a false interpretation at the species level. Many available popular pipelines nowadays like VSEARCH (Rognes et al. 2016), QIIME (Caporaso et al. 2010), Mothur (Schloss et al. 2009), etc., which can be used for fungal classification, also apply this algorithm making it more compatible with short-read sequencing technologies. Despite the enormous advantages of long-read NGS, most current metabarcoding studies only identify potential disease agents at the genus level and lack any definitive species identification due to the noisy-read nature of the sequence, making it hard to determine whether the sequence classified was the true species identity or just the artifact or the incorrect closely related taxa.

Uniform Manifold Approximation and Projection (UMAP) (Armstrong et al. 2021) and Hierarchical Density-Based Spatial Clustering of Applications with Noise (HDBSCAN) (McInnes et al. 2017) are two well-known tools used for clustering high-complexity information that can be used together in a two-step process for data analysis. First, UMAP can be used to reduce the dimensionality of high-dimensional data and project them into a lower-dimensional space, which can help to visualize the data and identify patterns or structures. Then, HDBSCAN can be applied to reduced-dimensional data to identify clusters based on their density and spatial proximity in the reduced-dimensional space, especially for noisy and complex data, such as reads obtained by nanopore sequencing. The combined approach, using UMAP for dimensionality reduction and HDBSCAN for density-based clustering, can help to identify meaningful clusters in complex datasets and facilitate further analysis and interpretation of the data. There is already a bioinformatic tool that applied the aforementioned tools for the identification of bacterial 16S rRNA nanopore sequencing datasets, which is called NanoCLUST (Rodríguez-Pérez et al. 2021). This tool has a high efficacy of identifying bacteria up to the species level. However, its implementation to fungal data sets and mixed species fungal data sets is still limited, because the pipeline is mainly aimed at bacterial identification.

In this study, our objective was to provide insights into the usefulness of nanopore sequencing for fungal species identification and to determine how the sequences generated from this technology should be presented and interpreted in terms of fungal identification at the species level. Specifically, we sequenced and analyzed eight individual yeast isolates using targeted sequencing of the full ITS region (ITS1, 5.8S, ITS2) to assess the accuracy of species identification from nanopore reads. Additionally, we simulated a scenario of mixed species reads, incorporating variations from each individual, to further test the accuracy of species identification using nanopore sequencing. We display the scenario of the identification of fungi at the species level using both pure isolate and mixed species datasets with different approaches for classification using nanopore raw reads directly, performing read correction and assembly with Canu (Koren et al. 2017), clustering the sequences using VSEARCH (Rognes et al. 2016), and clustering the sequences using the modified version of NanoCLUST to make it more compatible for fungal classification. We demonstrate fungal identification at the species level with three methods, both for pure isolate and mixed species identification. As a result, we propose a pipeline for species identification and the criteria to determine the species from either individual isolate reads or mock mixed species reads, in the case of mixed infections, with in our case three species simulating the cases of a mixed fungal infection in humans (Ahmadikia et al. 2021; Gülmez et al. 2020; Soll 2002; Teng et al. 2022), using nanopore reads.

## MATERIAL AND METHOD

### DNA extraction and amplification of the full-length ITS region

The genomic DNA (gDNA) was extracted from eight yeast strains from six species previously identified by PCR amplification and Sanger sequencing of the ITS1 and ITS2 regions (*Candida albicans* (n = 2), *Candida tropicalis* (n = 1), *Nakaseomyces glabratus* (formerly *Candida glabrata*) (n = 1), *Trichosporon asahii* (n = 2), *Pichia kudriavzevii* (formerly *Candida krusei*) (n = 1), *Cryptococcus neoformans* (n = 1)), which had been isolated from clinical samples from eight patients (see Additional file 1: Table S1). The gDNA was extracted starting from a culture derived from a single colony using an in-house lysis buffer (0.2 M NaCl, 0.02 M EDTA, 0.04 M Tris, 0.5% w/v SDS, and 0.5% v/v β-mercaptoethanol) and phenol–chloroform extraction. The extracted gDNA was then eluted with 40 µl nuclease-free molecular-grade water. This gDNA was quantified on the NanoDrop™ (Thermo Fisher Scientific, Waltham, USA). The extracted DNA was portioned into two aliquots prior to amplification. One aliquot was used for low-throughput Sanger sequencing to be used as a control to reidentify and confirm the species, and the other one was used for nanopore sequencing. The entire ITS region of all isolates was amplified with primers ITS1 (5′ TCCGTAGGTGAACCTGCGG 3′) and ITS4 (5′ TCCTCCGCTTATTGATATGC 3′) as previously described (37). The amplicons were purified using the QIAquick™ PCR Purification Kit (Cat. No. 28104, Qiagen, Maryland, USA), following the manufacturer’s protocol. The purified PCR products were quantified by Promega Quantus™ Fluorometer (Cat. No. E6150, Promega, Madison, USA), converted into femtomolar using NEBioCalculator v1.14, and adjusted the 100 fmol before proceeding to sequence.

### Nanopore sequencing

The gDNA libraries were prepared using the Ligation Sequencing Kit (cat. no. SQK-LSK109, Oxford Nanopore Technologies (ONT), Didcot, UK), as per the manufacturer’s protocol with the multiplexing steps using Native Barcoding Expansion 1–12 (cat. no. EXP-NBD104, ONT, Didcot, UK). This allows for the multiplexing of up to 12 samples per sequencing run per flow cell. Each yeast strain was tagged with a different barcode as shown in Additional file 1: Table S1. The DNA library was then loaded into the flow cell as per the manufacturer’s protocol.

**Fig. 1:**
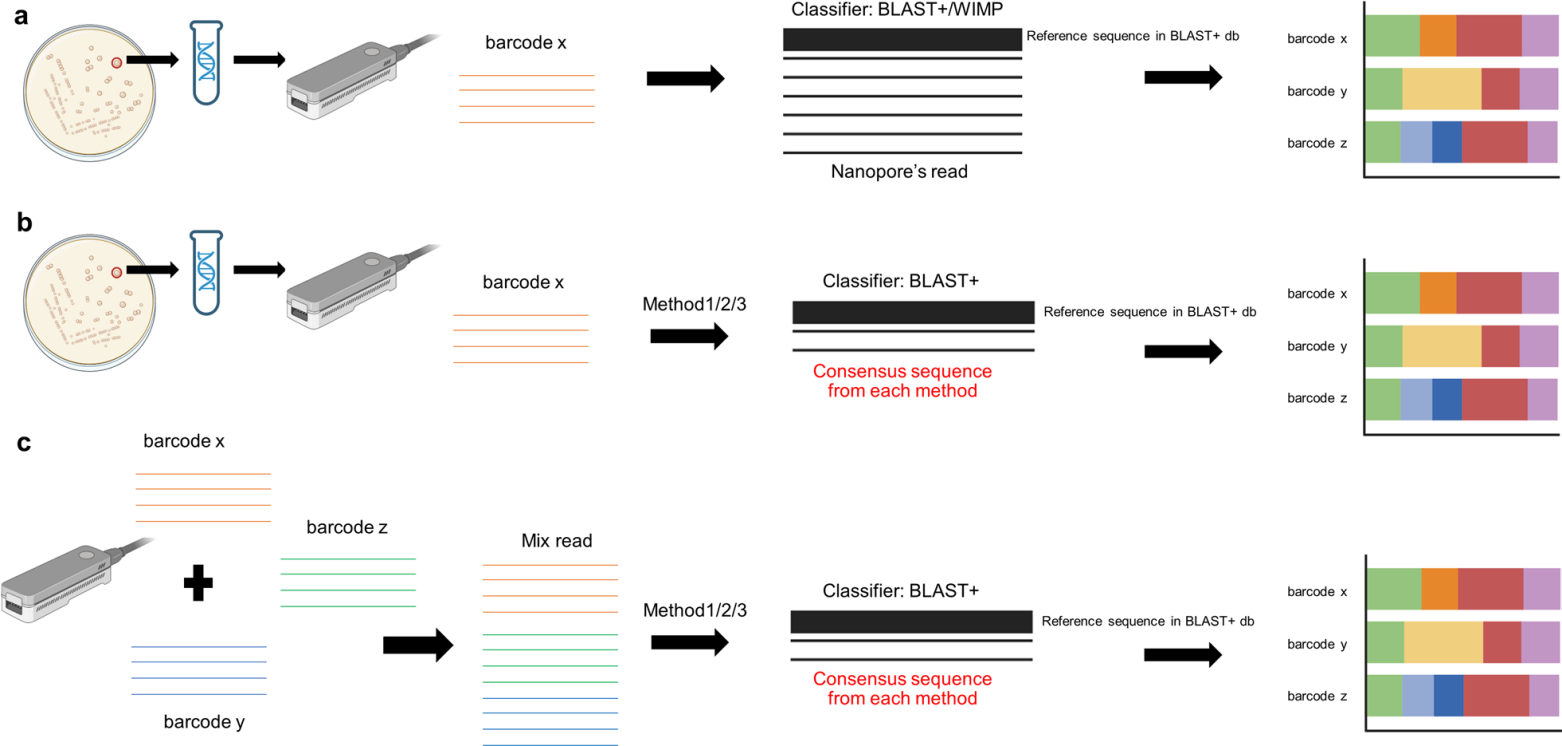
Diagram displaying the analytical steps performed in this study. **a** classification of raw data of ITS sequence from a single yeast colony obtained by Oxford nanopore sequencing with BLAST+ and WIMP from EPI2ME™ pipeline, **b** classification of consensus sequence generated with a different method of ITS sequence of single yeast colony obtained by Oxford nanopore sequencing with BLAST+ , and **c** classification of sequences of the different mix read scenarios generated by concatenated the sequence of a different yeast strain in various scenarios from the consensus sequence generated with a different method

The sequencing was performed using the MinION™ flow cell R9.4.1 (ONT, Didcot, UK). Sequencing preparation steps were followed according to the manufacturer’s protocol facilitated with the MinKNOW graphic user interface (ver. 21.11.8) to help set up the sequencing run without using the command line. Base-calling, adapter trimming, and demultiplexing were executed with Guppy (ver. 5.0.16, ONT) and bases were called using the super accuracy mode (the r9.4.1_450bps_sup_model provided by ONT with the preset expected accuracy per read equal to 98.3% in the speed of 0.06 Gbps/ hour using GPU base calling, 4 GB RAM according to manufacturer’s instruction), and the minimum Phred quality score (q-score) was set to equal to 15, to produce the raw Fastq files. The commands used for base calling are detailed in Additional file 2: Table S2. The Fastq files from each barcode were quality-filtered using Filtlong (ver. 0.2.1) with a quality filtering q-score of 20 (expecting the read accuracy of about 99% after filtering). The throughput and number of passed reads per barcode are shown in Additional file 3: Figure S1. The overview of downstream analysis is given in Fig. 1.

For Sanger sequencing, the purified amplicons were sequenced by single-pass DNA sequencing using an ABI 3730 genetic analyzer according to the manufacturer’s instructions. These results were used as controls.

### Read simulation in mixed species

Three scenarios of mixed species reads based on the number of reads per barcode (see Additional file 3: Figure S1) were generated using the filtered Fastq files of each barcode. First, reads from different genera and species: *N. glabratus* (*C. glabrata*)*, T. asahii*, and *C. tropicalis* (barcodes 1, 2, and 6, respectively) were concatenated together: mixed read scenario 1*.* It consisted of reads that were different in abundance. Second, reads from different species in the same genus: *P. kudriavzevii* (*C. krusei*), *C. tropicalis,* and *C. albicans* (barcodes 3, 6, and 8, respectively) were concatenated together: mixed read scenario 2. This scenario consisted of reads with a similar number of reads per barcode. Third, reads from different species in the same genus: *N. glabratus, C. albicans, and C. tropicalis* (barcodes 1, 5, and 6, in order) were concatenated together: mixed read scenario 3*.* This scenario consisted of reads with a different number of reads per barcode. The information of all mixed read scenarios is summarized in Additional file 4: Figure S2.

### Species identification directly from nanopore’s raw data

The filtered Fastq files proceeded to species identification using BLAST+ (version 2.13.0) (Camacho et al. 2009) which is the command line version of NCBI’s BLAST against NCBI’s Nucleotide database and EPI2ME™ using the WIMP pipeline to give the comparison of different classification pipeline. For the identification with BLAST+, each read was classified only when the highest score was hit from the BLAST+. The command line used for classification is given in Additional file 2: Table S2. The default setting of EPI2ME™ was applied. The results were shown by the stacked bar chart displaying the species composition classified from the reads and the standard output in the case of EPI2ME™. The workflow for species identification from nanopore’s raw data of the isolates is displayed in Fig. 1a.

### Species identification with read correction and assembly with Canu

**Fig. 2:**
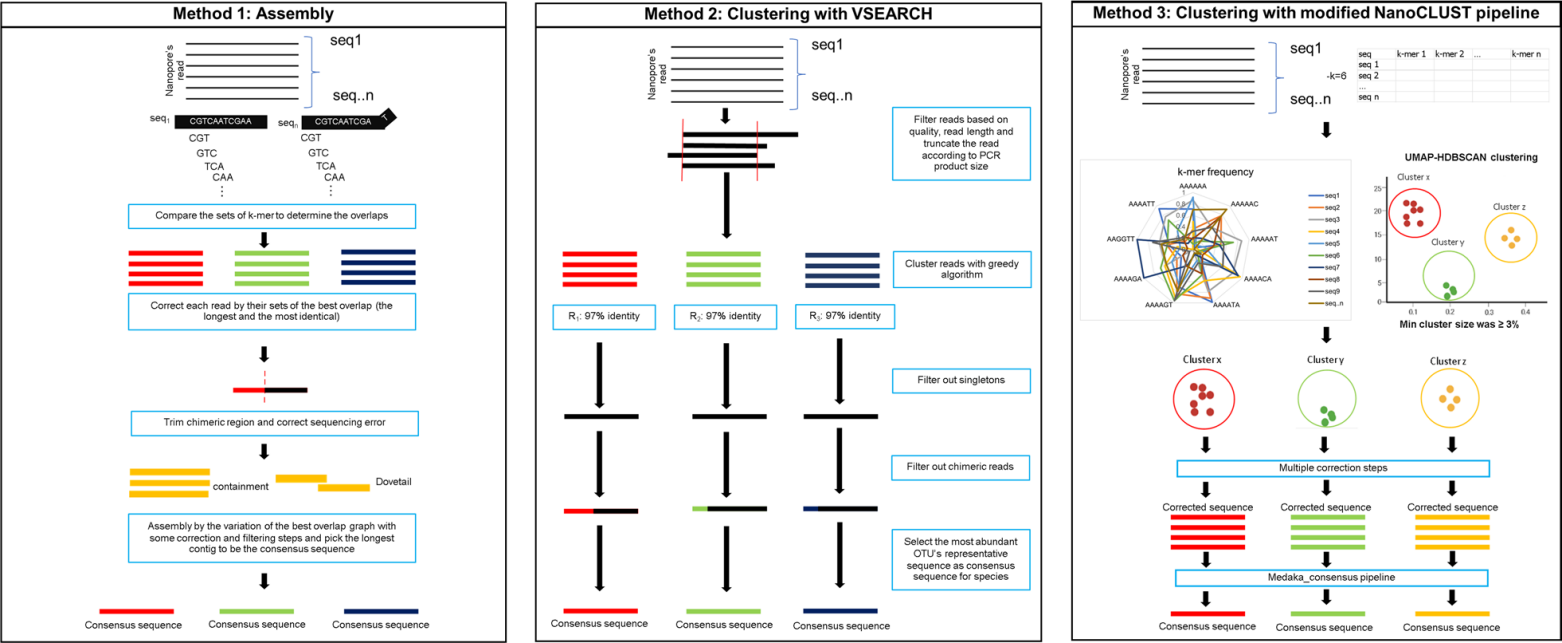
Methods for generating consensus sequence for single isolate and mix-species read identification. (**Method 1**) performing read correction and assembly with Canu, **(Method 2)** clustering the sequences using VSEARCH, and **(Method 3)** clustering the sequences using a modified version of NanoCLUST

The quality-filtered Fastq files of both individual and mixed barcodes proceeded to the correction and assembly steps using Canu (Koren et al. 2017). The command line argument given to the program is provided in Additional file 2: Table S2. The longest contig produced from the program was chosen to be the consensus sequence and proceeded to the species classification steps using BLAST+ as mentioned above in the case of the fungal isolate’s classification. In the case of reads from mixed species, all the contigs generated proceeded to the BLAST+ search. The workflow for species identification from the isolates and reads from mixed species is displayed in Fig. 1b, c, respectively. The correction and assembly steps are referred to as Method 1 in Fig. 2.

### Species identification from reads clustered with VSEARCH

The quality filtered Fastq files of each barcode and the mixed reads proceeded to the correction and assembly steps using VSEARCH. The command line argument given to the program is provided in Additional file 2: Table S2. In brief, the software performed OTU clustering in a centroid-based fashion. First, the sequence was trimmed based on its amplicon length. After that, the query sequence was initially selected to be the “reference” sequence. This reference sequence will be used as the template for the other reads to compare. The sequences were grouped in the same cluster if they shared a similarity of ≥ 97%. If there are sequences that did not cluster with the existing group, they become the reference sequences for new groups and the grouping continues until there is no sequence left to be assigned to any group. The sequences that have not clustered in any group or the ungrouped reads (singleton) were discarded. The grouped reads proceeded to the chimera filtering steps according to the default setting of VSEARCH (Rognes et al. 2016). All “reference” sequences from all OTUs then proceeded to BLAST+ search to show the species classification of all assigned OTUs. The result was shown in a stacked bar graph showing the species composition in percentage and in a dot-plot showing the OTUs and abundance of supportive sequence per OTU for both with and without filtering singleton (OTU with only one sequence as the member of that OTU) steps. For reads from mixed species, OTU clustering was performed as mentioned with the steps of singleton filtering, the result is also shown as a stacked bar graph showing the species composition in percentage and a dot-plot showing the OTU and abundance of supported sequences per OTU. The workflow for species identification from the isolates and reads from mixed species is displayed in Fig. 1b, c, respectively. The overview of this pipeline is shown in Method 2 of Fig. 2.

### Species identification using a modified NanoCLUST pipeline

The quality filtered Fastq files were used as the input for running the modified version of the NanoCLUST (Rodríguez-Pérez et al. 2021) pipeline with the executable file available in Python and shell script namely ont_cluster.py and umap_consensus.sh respectively. The code snippets were available via GitHub and GitLab. This pipeline was modified from the original NanoCLUST in some parameters, output format, and some correction steps to make it more compatible with analyzing the fungal ITS datasets included in this study. Significantly, this modified version involves setting the filtering threshold for the clustered sequence at 3%, meaning that a sequence must have a read count greater than 3% compared to all available reads in the dataset to proceed to the next correction and consensus-sequence generation steps. The modifications made to the NanoCLUST pipeline were limited to the consensus sequence calling steps. The command line argument given to ont_cluster.py is shown in Additional file 2: Table S2. After the consensus sequence was generated, all the passed consensus sequences proceeded to the classification steps with BLAST+in both individual species identification and mixed species read identification. The results were shown as the HDBSCAN clustering results with the identification results of the consensus sequence from all the pass clusters. The workflow for species identification from the isolates and reads from mixed species is displayed in Fig. 1b, c, respectively. The overview of this pipeline is shown in Method 3 of Fig. 2.

Our study also included testing the pipeline with subsampling reads of not more than 10,000 reads both for individual isolate reads and reads from mixed species of all scenarios. The subsampling was done using seqtk (Shen et al. 2016) to test whether the composition of the identified species will change with a lower number of reads. This process was aimed to facilitate the pipeline analysis for the condition of lesser reads, and to reduce the computer workload for analyzing sequencing reads for species identification from large data sets.

## RESULTS

### Read correction and assembly with Canu successfully generated the consensus sequence for the species identification of individual yeast isolates but failed to identify the reads from mixed species

**Fig. 3:**
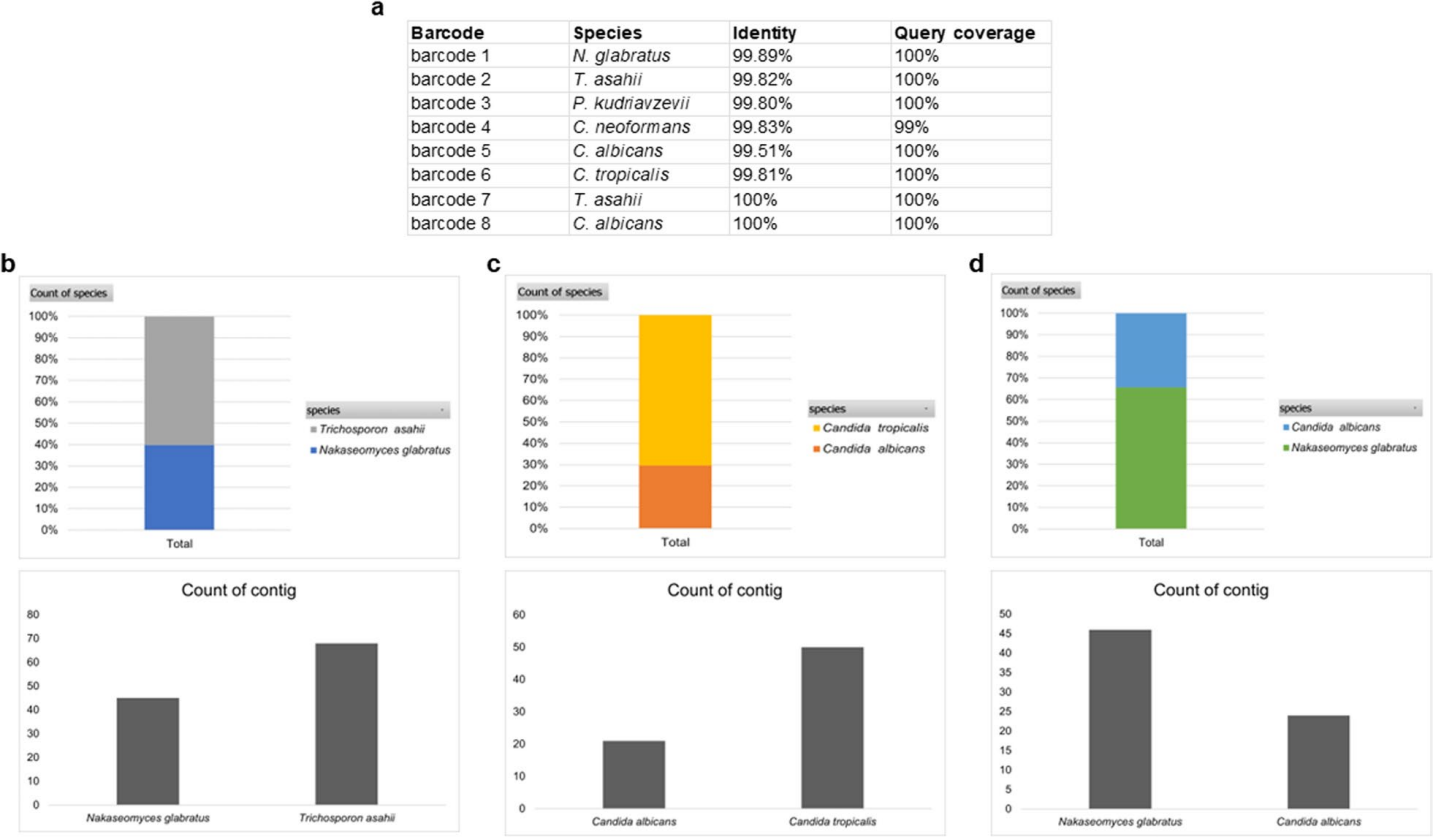
Classification results using the consensus sequence generated by Canu, showing the identified species together with percent identity and query coverage obtained from BLAST+ search using the longest contig chosen as consensus sequence. **a** identifying an individual isolate, **b** the percentage of species identified from all contigs obtained from the mix-species reads of mix reads scenario 1, **c** percentage of species identified from all contigs obtained from the mix-species reads of mix reads scenario 2, and **d** percentage of species identified from all contigs obtained from the mix-species reads of mix reads scenario 3 (see Materials and Methods)

All eight yeast isolates were successfully identified to the species level using the correction and assembly with Canu with an overall percent identity of more than 99% and query coverage of 100% (Fig. 3a). However, in the case of the mixed read scenario, Canu failed to recall some of the species mixed in the mixed read in all scenarios as it missed out on the lower abundant species and only called two out of three species in all scenarios (Fig. 3b–d).

### Species identification of nanopore’s raw data using BLAST+ showed better results than WIMP and revealed that the reads projected from the nanopore sequencing were not completely homogenous

**Fig. 4:**
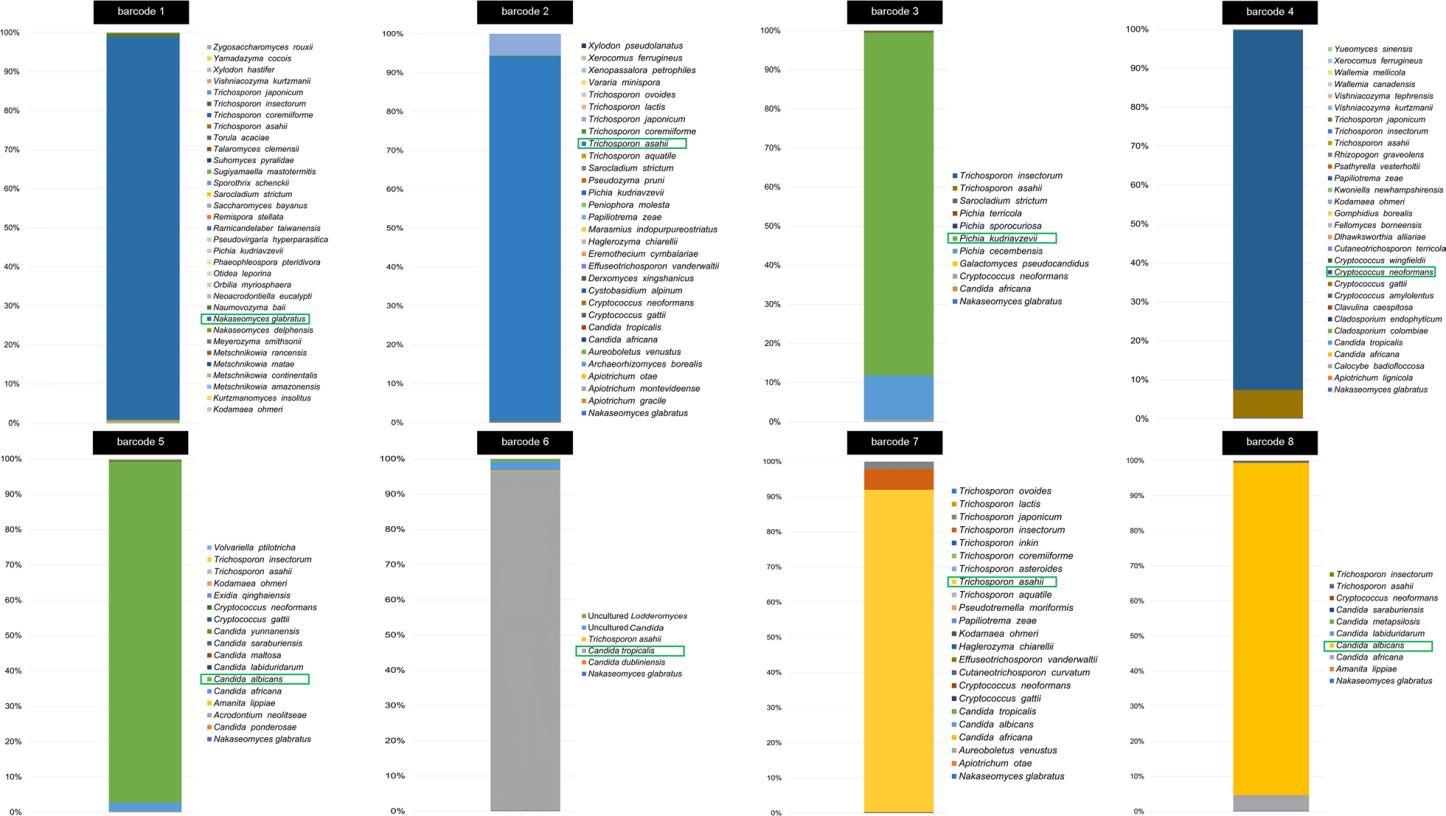
Classification results of nanopore raw sequence data using BLAST+ , species composition in percentage assigned for each barcode using BLAST+ for species classification. The green box indicates the true species of each barcode

The nanopore raw data were analyzed using BLAST+to identify yeast species based on ITS barcode reads. However, the results showed that the ITS barcoded reads did not exclusively represent only one yeast species, as they were classified into multiple species instead of just one. All barcodes were classified into their true positive species including *N. glabratus* for barcode 1, *T. asaihii* for barcode 2, *P. kudriavzevii* for barcode 3, *C. neoformans* for barcode 4, *C*. *albicans* for barcode 5, *C*. *tropicalis* for barcode 6, *T. asahii* for barcode 7, *C*. *albicans* for barcode 8 and other taxa as shown in Fig. 4. It is also worth mentioning that the false positive identification from BLAST+raw data did not exceed 15% (Fig. 4) in all cases indicating that the possible error from the long-noisy read nature of nanopore reads only has a minimum effect on BLAST+classification. On the other hand, classification results using WIMP in all cases resulted in false identifications, with not even closely related taxa being suggested for some of the barcodes (Additional file 5: Figure S3).

### Read clustering using VSEARCH can correctly classify the species of both individual yeasts and mixed read scenarios with specific criteria

**Fig. 5:**
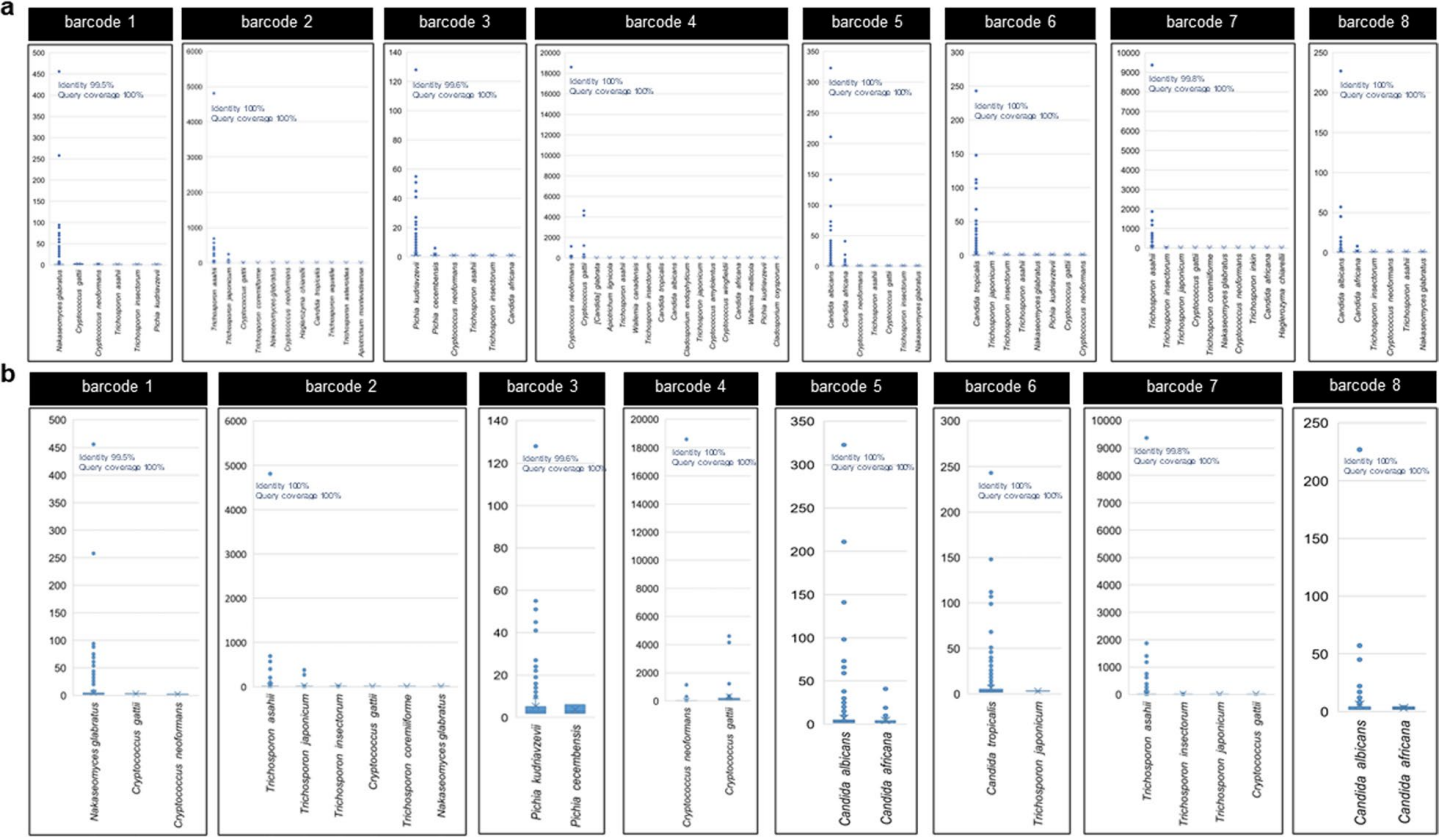
The abundance of each OTU per classified species. **a** abundance of each OTU (the value of the dot) per classified species projected from VSEARCH in case of analyzing with yeast strain not including singleton filtering and identification results showing percent identity and query coverage of most abundant OTUs representative sequence, and **b** abundance of each OTU (the value of the dot) per classified species projected from the VSEARCH in case of analyzing with yeast strain including singleton filtering steps and identification results showing percent identity and query coverage of most abundant OTUs representative sequence

**Fig. 6:**
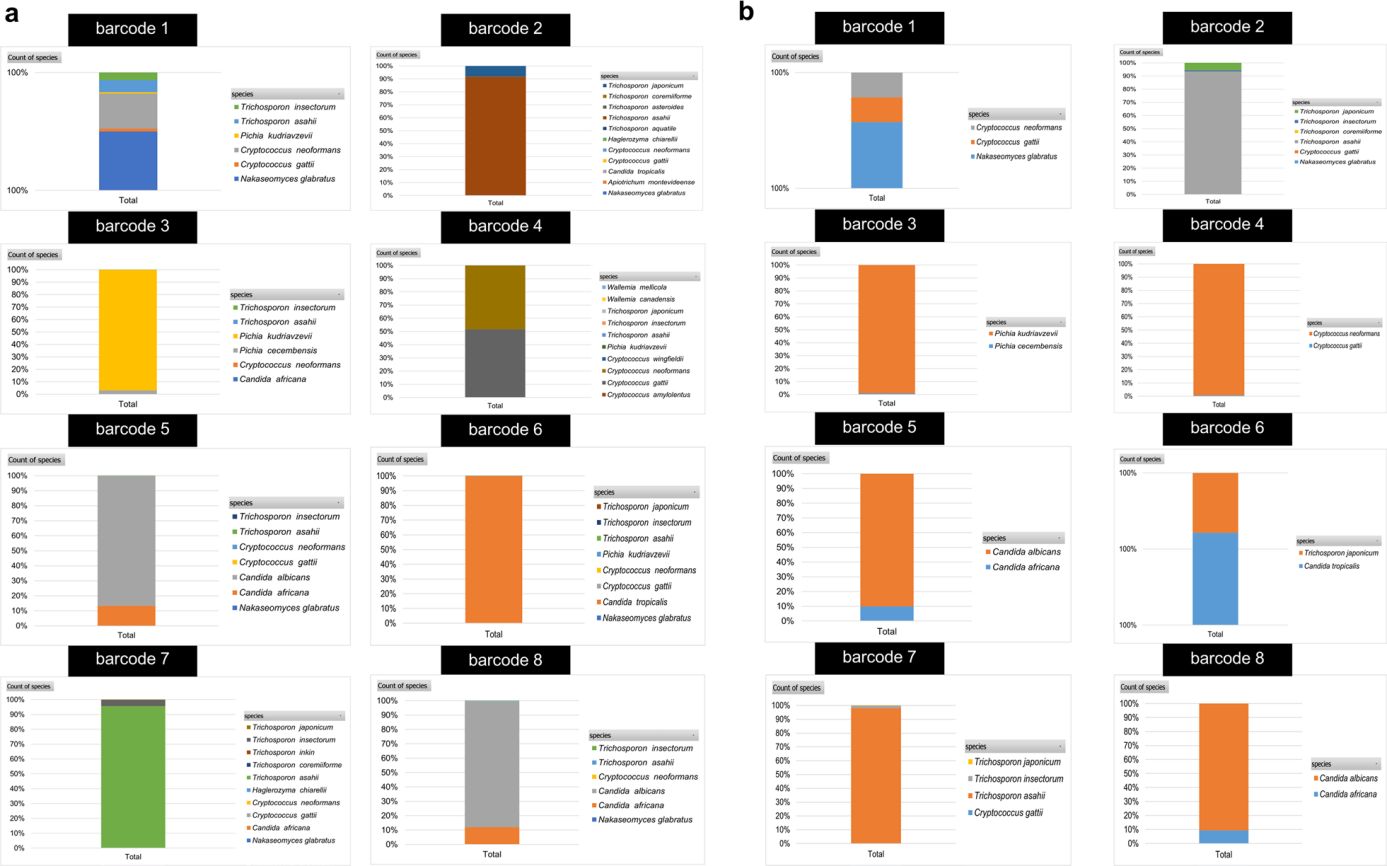
Classification results of the representative sequence of each OTU clustered by VSEARCH of individual isolates. **a** Identification results showing the percentage of species identified from all representative sequences projected from the program analyzing individual isolates not including singleton filtering steps, and **b** identification results showing the percentage of species identified from all representative sequence projected from the program in case of analyzing individual isolates including singleton filtering steps

By clustering the sequences based on similarity using VSEARCH, it was found that with the 97% cut-off identity, the results showed a large number of OTUs, as is expected from clustering long-noisy reads based on similarity (Fig. 5 and Additional file 6: Figure S4). Strikingly, the species composition classified via identification of the representative sequence from the OTU found many noteworthy points. First, for the species identification level, it was found that the most abundant OTUs representative sequence was always classified as the true positive species and was given a percent identity of more than 99% and a query coverage of 100% in all cases (Fig. 5a, b). Second, the true positive species always have the largest number of OTUs (Fig. 6a, b). Third, the false positive species were mostly from a singleton OTU, which is usually filtered out in the clustering steps (Figs. 5b and 6b). It also was found that when filtering out the singleton, the false positive OTU remains lower than 10% of all classified OTUs (Fig. 6b).

**Fig. 7:**
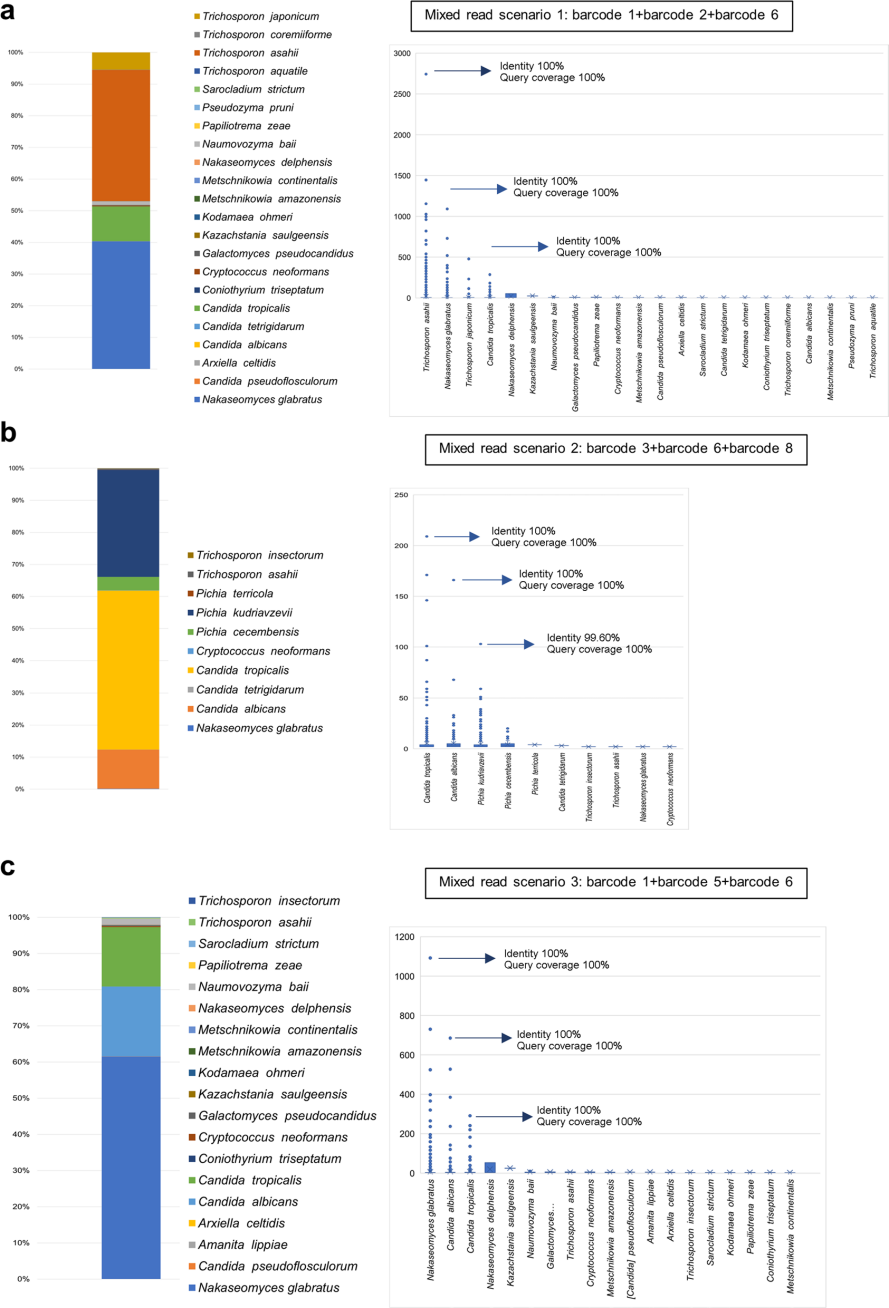
Classification results of the representative sequence of each OTU clustered by VSEARCH of mix-species reads. The stacked bar graph displaying the percentage of species identified from all representative sequences from OTUs projected from the program including singleton filtering steps and the dot plot displaying the number of OTUs and abundance of each OTU per classified species projected from the program analyzing mix-species reads: **a** mixed read scenario 1, **b** mixed read scenario 2, and **c** mixed read scenario 3

For detecting mixed species samples, a threshold of 10% OTU abundance was used to rule in the species to be classified as true positive species, and the most abundant OTUs representative sequence of the rule-in species was used as consensus sequence to identify the species using BLAST+, mirroring the approach used for individual yeast identification. It was found that these criteria resulted in filtering out the false positive OTUs from the analysis (Fig. 7a–c). The identified species that passed the criteria included *T. asahii*, *N. glabratus,* and *C*. *tropicalis* for the mixed read scenario 1 (Fig. 7a), *P. kudriavzevii*,* C*. *tropicalis,* and *C. albicans* for the mixed read scenario 2 (Fig. 7b) and *N. glabratus*, *C*. *albicans* and *C*. *tropicalis* for the mixed read scenario 3 (Fig. 7c), respectively. The rule-in species’ most abundant OTUs representative sequence was used as the consensus sequence to identify the species and give the correct identification results with a percent identity of more than 99% and a query coverage of 100% in all mixed read scenarios (Fig. 7a–c). This indicated that these criteria can be used to identify mixed species despite factors: like read abundance (which in this study has the lowest reads of less than 5,000 reads in the case of barcode 8), amplicon length, or presence of species within the same genus in case of mixed species of not more than three species.

### Read clustering using a modified NanoCLUST pipeline can effectively classify the species of both individual yeasts and the mixed read scenarios

**Fig. 8:**
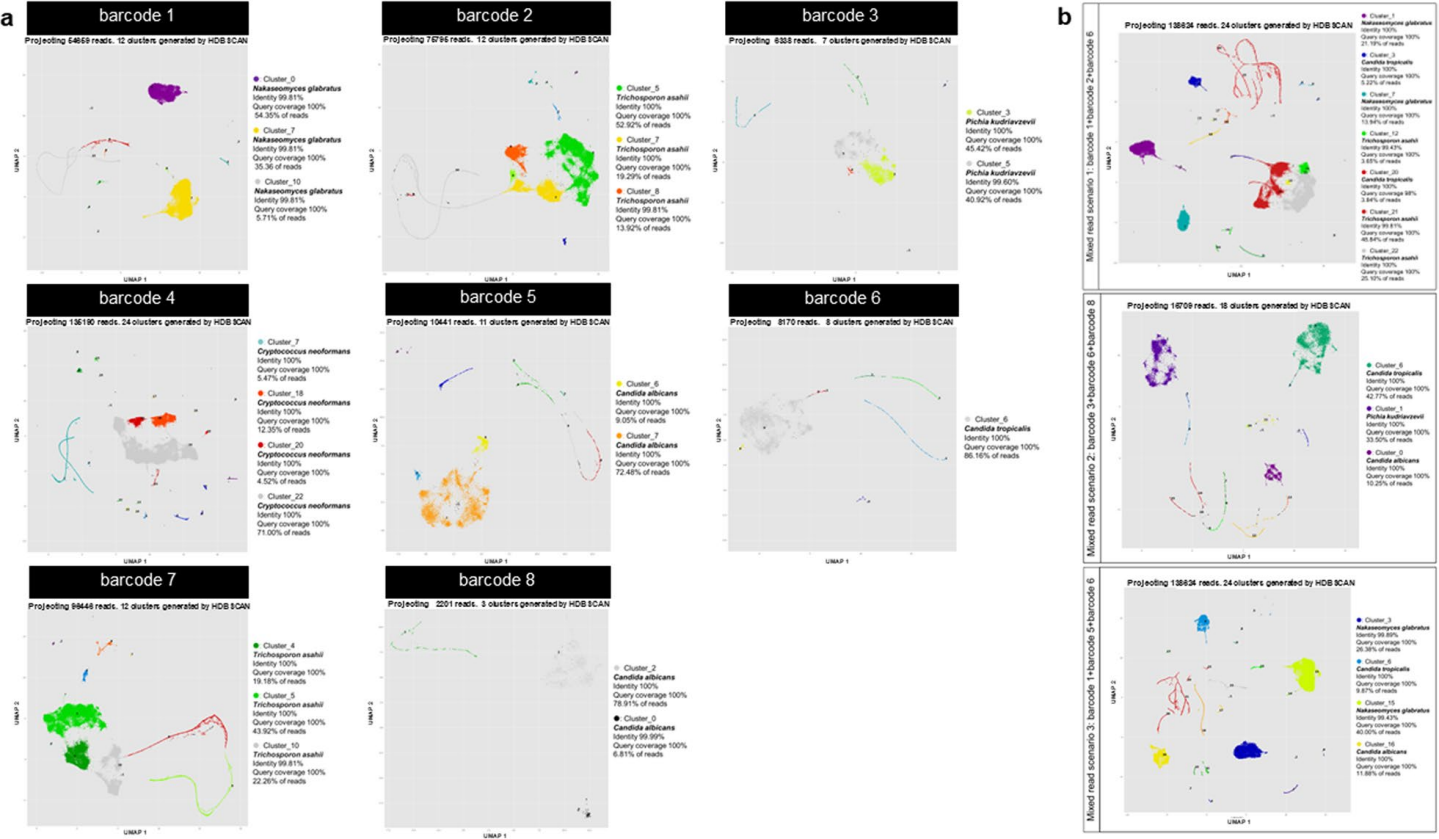
Clustering and classification results of the consensus sequence of each cluster generated by the modified NanoCLUST pipeline from individual isolate and mix-species reads. The Cluster plot generated with ggplot showing the clustering result from modified NanoCLUST pipeline with the identification information of the consensus sequence from the pass cluster showing species, percent identity, query coverage getting from classification with BLAST+ , and the percentage of reads assigned to the following cluster for **a** individual isolate reads and **b** mix-species reads from different mix read scenarios

**Fig. 9:**
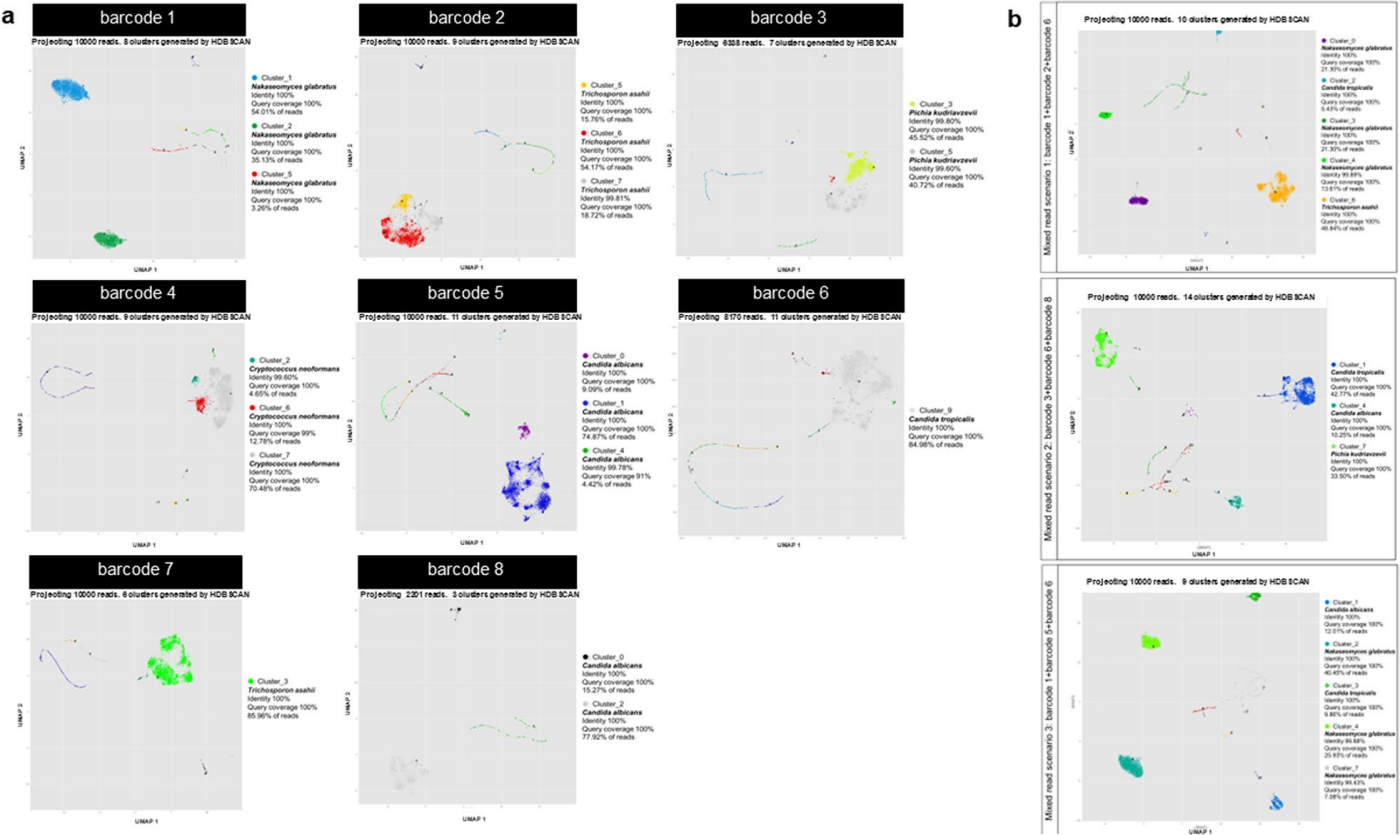
Clustering and classification results of the consensus sequence of each cluster generated by the modified NanoCLUST pipeline from individual isolate and mix-species reads from different mix read scenarios with the subsampling datasets. The Cluster plot generated with ggplot showing the clustering result from the modified NanoCLUST pipeline with the identification information of consensus sequence from the pass cluster showing species, percent identity, query coverage getting from classification with BLAST+ and also the percentage of reads assigned to the following cluster for **a** individual isolate reads, and **b** mix-species reads from different mix read scenarios

The program herein proposed uses UMAP-HDBSCAN clustering to cluster the sub-sequence (k-mer) profile from each nanopore read, followed by read density filtering and multiple steps of read correction to generate consensus sequences for each cluster. The consensus sequences correctly identified the species for each barcode (single yeast strain) (Fig. 8a), including *N. glabratus* for barcode 1, *T. asahii* for barcode 2, *P. kudriavzevii* for barcode 3, *C. neoformans* for barcode 4, *C*. *albicans* for barcode 5, *C*. *tropicalis* for barcode 6, *T. asahii* for barcode 7, *C*. *albicans* for barcode 8, as well as the mixed species reads in all scenarios (Fig. 8b). The consensus sequences have a percent identity of more than 99% and a query coverage of more than 98% in all cases (Fig. 8a, b). The number of classified clusters was reduced compared to clustering with VSEARCH (Additional file 6: Figure S4). The identification results of subsampling datasets showed no difference in the species name, percent identity, query coverage, % read, and several clusters compared to the full datasets (Figs. 8, 9). The consensus sequences generated by this pipeline have similar identification results with the full datasets, with an identity of more than 99% and a query coverage of more than 98% (Fig. 9a, b). The number of clusters in both conditions (full datasets and subsampling datasets) from both yeast strains and mixed species read identification varies by not more than three clusters in all cases (Additional file 6: Figure S4), and all additional clusters are identified as the same species present in the dataset (Figs. 8, 9).

## DISCUSSION

Next-generation sequencing (NGS) technologies, particularly second-generation sequencing methods, produce short reads with high throughput and exceptional accuracy. However, these technologies present challenges in species identification and variation analysis due to the limited length of the generated reads and the computational steps involved in the data processing. Short-read sequencing techniques typically require paired-end sequencing, wherein only specific lengths of DNA fragments are sequenced (approximately 150–250 bp. depending on the sequencing platform), and subsequent efforts are needed to join the paired reads and assemble them to obtain the complete sequence of longer amplicons. To accomplish such tasks, specific bioinformatic pipelines are often required with the complication of merging and tracking the reads pairs. Moreover, longer amplicon lengths typically necessitate more computational power and resources to process the increased number of reads. While user-friendly software with a coding-free interface is available for these analyses, they are often only commercially accessible. Consequently, the cost of establishing an NGS laboratory is further inflated, considering the expenses associated with the sequencing machine installation and maintenance. As an alternative for amplicon sequencing for pathogen identification purposes, nanopore sequencing has emerged as a promising alternatives to address these limitations. Nanopore sequencing offers high-throughput DNA sequencing and captures single reads in their full length as the benefit from long-read single molecule sequencing. It also presents advantages such as smaller instrument size, ease of use, the potential for near real-time analysis on-site, and lower installation and maintenance costs compared to other sequencing platforms. However, nanopore sequencing is associated with a higher error rate which necessitates careful differentiation between biological variations and technical errors within the generated sequence data. A specific problem with the reads generated by nanopore sequencing is the case of sequencing-induced indels in reads (Mikheyev and Tin 2014). Although several solutions exist to alleviate this problem, like the more developed chemistry (presently, the updated flow cell is R.10.3) designed to specifically address this problem, with the additional requirement of more input DNA, resulting in less sequence output, which however is only compatible with few of the available sequencing kits or quality filtering. Further developments may need to be applied to solve this problem (Delahaye and Nicolas 2021). The inherent noisiness of reads generated by the nanopore sequencing technology requires careful consideration when interpreting species based on representative sequences obtained through processes like sequence clustering or assembly.

Therefore, our study aimed to visualize and evaluate the representative sequences generated from nanopore reads for identification. The first approach examined used the read correction and assembly software, Canu. This program works by including steps of self-correction (without using a reference), which are based on the comparison of the sketch of the k-mer of the given query reads to generate the overlap between the reads. Then, it assigns some of the best overlaps (usually the longest) to give the information to correct each read (Koren et al. 2017). This step continues in multiple rounds until a consensus sequence is generated. It also uses the specific k-mer weighting algorithm to resolve repeat or deletion that occurred due to a technical error in the single-molecule noisy read to select the k-mer from each read to generate the overlap (Berlin et al. 2015). This step tends to also alleviate the effect of the homopolymer-induced error (Delahaye and Nicolas 2021) which is the typical error found in nanopore reads. The results of our study showed that correction and assembly with Canu successfully generated the consensus sequence for species identification of individual yeast isolates, with an overall identity of more than 99% and a query coverage of 100% in all isolates. However, Canu failed to identify mixed species reads accurately, as it missed out on lower abundance species and only called two out of three species in all scenarios. This result shows that this method can help to facilitate the identification in connection with the use of other tools but cannot be used for identification alone.

Species identification of nanopore's raw data using BLAST+ showed better results than WIMP. The barcoded reads, which were supposed to represent reads from only one yeast isolate, were classified into true positive species and their closely related taxa in all cases. The false positive identification from BLAST+ was found to be minimal, not exceeding 15%, despite the homopolymer errors from the long-noisy nature of nanopore reads. On the other hand, classification results using WIMP were found to be false identifications, with no closely related taxa for some barcodes. This situation could be explained by the principle of these two classifiers, while BLAST+ uses the seed extension of the k-mer length of 11, using dynamic programming for determining the local alignment, the WIMP pipeline, which implemented Centrifuge (Kim et al. 2016). It searches for regions in the sequence that exactly match the database (no-gap alignment) and uses these matches for classification. However, this approach may not be fully compatible with the long and noisy reads generated by ONT sequencing that could obstruct the matching and interpretation of matching results.

Read clustering using VSEARCH with a specific condition of 97% identity cutoff effectively classified the species of both individual and mixed read scenarios. Clustering based on similarity resulted in a large number of OTUs, as expected from clustering long-noisy reads. The analysis of representative sequences from the OTUs revealed firstly that the most abundant OTUs representative sequence is always classified as the true positive species, with more than 99% identity and 100% query coverage; and secondly that the true positive species always had the largest number of OTUs. These phenomena indicated that for the species that are the true positive species in the datasets, most of the reads should be identified into that species. Therefore, the false positive species were mostly from singleton OTUs, which are usually filtered out in the clustering steps. Filtering out the singletons resulted in the fact that false positive OTUs remained lower than 10% in all cases. As such, this cut-off of 10% shall be used in the case of clustering by this principle (clustering in centroid-based clustering depends on the similarity of sequence) for determining the true positive OTU to be selected for further species identification.

For mixed species identification, a criterion of considering species with OTUs of more than 10% was applied, like the results found for individual identification. These criteria filtered out false positive OTUs from the analysis. Using the consensus sequence of the most abundant OTUs representative sequence as the identification sequence resulted in correct identification with more than 99% identity and 100% query coverage in all mixed species scenarios, indicating that this criterion should be used to identify mixed species despite such factors like read abundance, amplicon length, or the presence of species within the same genus in case of mix species not exceeding three species.

However, read clustering using a modified NanoCLUST pipeline was found to effectively classify the species of both individual yeasts and mixed species read scenarios with higher sensitivity than VSEARCH, especially for complex and noisy data such as the nanopore reads. Clustering based on k-mer profile comparison of each read followed by UMAP-HDBSCAN clustering and read density filtering of 3% resulted in correct species classification for individual isolate reads and mixed species reads in all scenarios. The number of classified clusters is reduced compared to clustering with VSEARCH (Additional file 6: Figure S4) making the identification steps with BLAST+faster. The cut-off abundance for true positive cluster determination was also lower than clustering using sequence similarity alone like VSEARCH (3% compared with 10% from VSEARCH). Furthermore, our results demonstrated that subsampling the sequence data by no more than tenfold while generating visualizations still yielded similar identification results. This suggests that our analytical pipeline could potentially benefit from reduced computational requirements without compromising reliability, particularly in cases where the dataset or infection scenario involves fewer pathogenic species in a mixed fungal infection (Soll 2002). However, for complex datasets like in mycobiome studies, the analysis of the full dataset is still recommended since the subsampling may cause of losing some taxa that have very low abundance in the datasets. It is also important to mention that the subsampling was done with the largest reduction of information being not more than 15-fold (as in the case of the barcode 4 was from 135,190 to 10,000 reads) for isolate identification and 15-fold (as in the case of mixed read scenario 1 with the reduction of 138,624 reads to 10,000 reads) with the largest difference of species mocked in the dataset of 9.27-fold (75,795 reads for barcode 2 and 8,170 reads for barcode 6). A larger reduction of information than this may affect the identification results in case of missing the low abundance taxa.

The modified NanoCLUST and VSEARCH are tools for clustering sequence reads, but modified NanoCLUST showed to be more effective for handling longer read lengths common in nanopore sequencing. It utilizes a k-mer profile comparing approach for clustering reads, which can more accurately cluster the long-noisy reads obtained by nanopore sequencing. Additionally, it was designed to handle high error rates and variations in read lengths found in nanopore sequencing and can handle large datasets efficiently. Moreover, it reduces the number of clusters and speeds up identification steps with classifiers like BLAST+. Finally, clustering based on the k-mer profile makes assigning sequences to each cluster more difficult resulting in reducing the chance of false positive identification. After clustering, the pipeline also consists of multiple correction steps before generating a consensus sequence from each cluster, allowing the identification of fungi for individual and mixed infection schemes up to the species level.

The modified NanoCLUST has great potential for identifying and differentiating various fungal species with high specificity and sensitivity. The pipeline can efficiently generate consensus sequences for each cluster, giving it the great potential not only to classify the species but also to be used for multi-locus sequence typing (MLST) studies or any other sequence type analysis and strain identifications. In metabarcoding, the pipeline improves accuracy and sensitivity by distinguishing between closely related species and detecting low-abundant species that may be missed out using conventional methods.

Overall, the results suggest that for fungal species identification using targeted sequencing of the ITS region with ONT, the modified NanoCLUST pipeline is an effective method for identifying fungal species from nanopore raw data both for the individual and mixed species identification, with the resolution in our case of three mixed species with the lowest read abundance for the species of more than 2,000 reads tested with these datasets. The findings from this study also provide valuable insights into the identification of fungal species in complex samples and contribute to the development of robust and accurate methods for the sequence data analysis generated by nanopore sequencing.

The objective of our study was to evaluate the software's ability to classify fungal pathogens from different datasets, considering factors such as the quantity of reads and the composition of fungal read in the datasets (see Additional file 4: Figure S2). As such we used ITS sequence reads from individually amplified strains to construct mixed species mock communities. We did not investigate the impact of experimental laboratory effects, such as PCR bias, sequence imbalances, and other constraints that arise during the experimental process. In the case of PCR bias and efficacy, it has previously been reported that a template concentration difference of up to tenfold affected the OTU clustering of ITS sequences depending on the species investigated, as it results in losing the less abundant species depending on the individual characteristic of the amplified sequences, like the length of the target region and the PCR success rate (Mafune et al. 2019). In a study looking into the identification of human pathogenic fungi, it has been shown that using different primer pairs, which generate different sizes of amplicons, has only a minimal effect on the number of classified sequences assigned to OTUs both for an individual strain and mock communities of 10 pathogenic fungi (Ohta et al. 2023). This study clearly showed that the relative abundance of individual classified species is not the same despite the equal amount of genomic DNA of each species in mocked DNA samples, suggesting that the bias arises as the specific characteristic of each species in a mock community with no correlation to the amplicon length or the primer pair used. However, it is still unclear whether the cause resulted from PCR bias or the grouping of some sequences since the similarity threshold was set quite low to include more sequences in the cluster. Although the read numbers of each species can be predicted to be less than 10,000 reads, it still is nearly impossible to determine the actual read number of each species in the mock community (Ohta et al. 2023). Moreover, these studies also used OTU clustering based on sequence similarity which is already aforementioned in this study to likely have less sensitivity than the UMAP-HDBSCAN clustering from NanoCLUST. Additionally, it is worth mentioning that there is currently a lack of peer-reviewed studies using the NanoCLUST (or similar tools) specifically for human pathogenic fungal classification, which address the limitations arising from PCR efficacy or differences in PCR success rates among various species within the region of interest, such as the ITS region. In addition, the fact that the sequence imbalance observed could potentially originate from the library preparation or sequencing steps has not yet been addressed. Therefore, it is crucial to further investigate these aspects through future benchmarking studies comparing them with other tools available.

### The proposed bioinformatics workflow

**Fig. 10:**
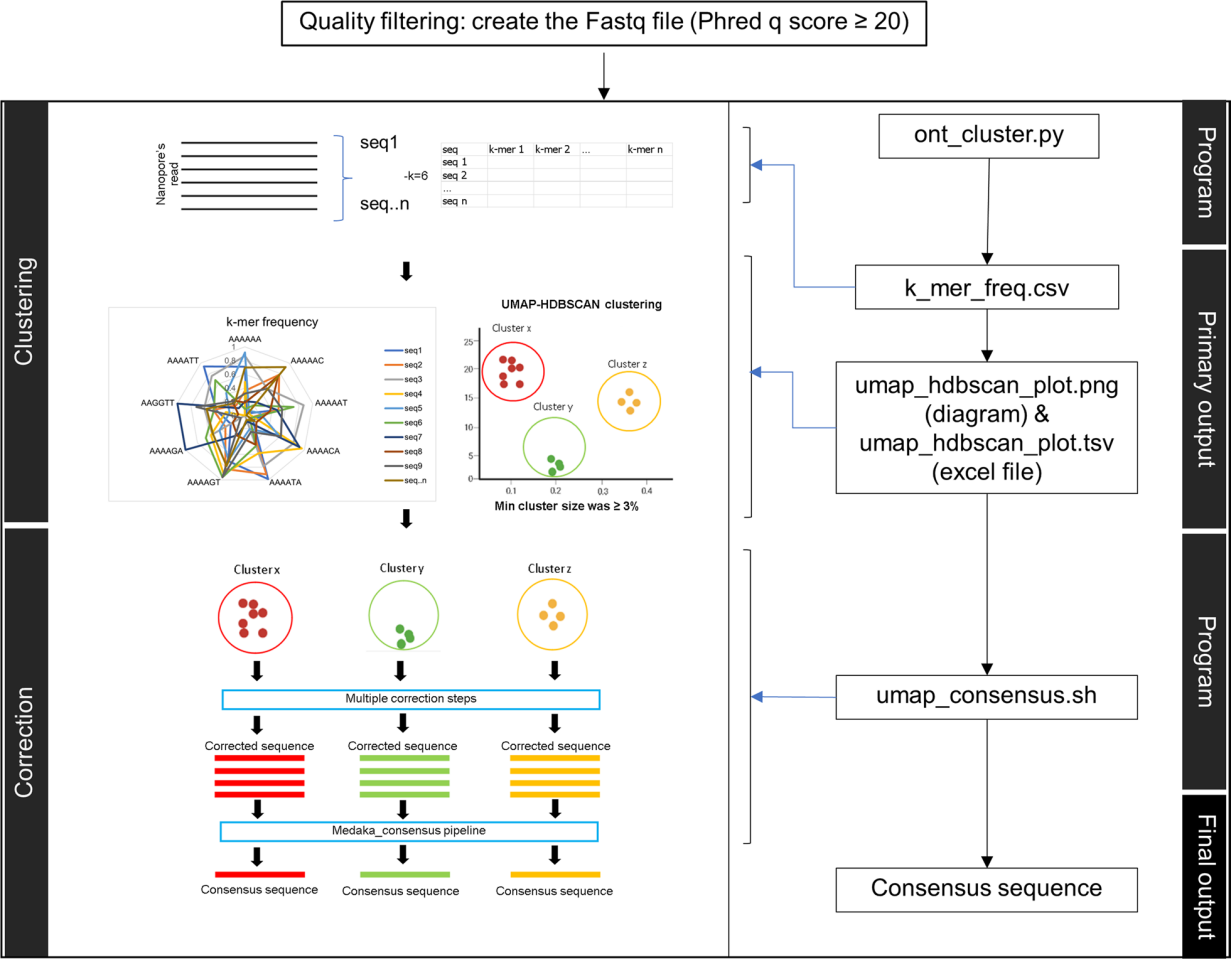
A proposed pipeline of the modified NanoCLUST for the generation of consensus sequence from ITS sequence obtained by nanopore sequencing for the species identification, both in principle (left box) and practical process (right box)

We propose herein a new bioinformatic workflow to analyze ITS sequences generated via nanopore sequencing (Fig. 10). Our workflow classifies the generated consensus sequence for each taxon presented in the dataset of each species. First, we performed a base call, trimmed the adapter, and demultiplexed the barcodes, by using the tools kit provided with the MinKNOW software and filtered the quality reads using a q-score of 20 to get an accuracy of around 99% per sequence. The filtered Fastq files generated are used as input for running the program ont_cluster.py which is the modified version of the NanoCLUST pipeline as it generates the k-mer frequency feature table and runs UMAP-HDBSCAN clustering. The output from the following program proceeded to the next program, which is umap_consensus.sh, which filtered the cluster, corrected sequences from the pass cluster, and generated the consensus sequence based on the corrected sequence that is the member of the assigned cluster. The final output of the program is the consensus sequence, which is the representative sequence of each pass cluster that proceeded to the further classification steps to identify the species. Our study tested the pipeline on a mixed species set of three species with the lowest number of reads per species of 2,000 reads, testing with the same and different amplicon lengths, and the same and different yeast species/genera. Further testing of more datasets with the newly developed pipeline will be needed to validate the pipeline in the future. Notably, the study included only a limited number of common pathogenic yeast species. More exotic pathogenic yeast species should be included together with testing of mold datasets in future studies.

## CONCLUSION

In conclusion, our work aimed to improve the sequence identification of pathogenic fungi using long-read sequencing. By using the herein developed sequence analysis pipeline, we will accumulate more information on the sequence variation within and between species, and enable the development of an interactive AI-like database by assigning the reference sequence as the ‘representative sequence’, accumulating the sequence types to create the population of the cluster with information of the distance among them, describing the boundary of distances between each population to discriminate different species by the distance between OTUs. In this way, the species will be predicted based on the homology, data density, and distance implementing most likely a neural network analysis.

Our study demonstrated the efficacy of targeted sequencing for fungal species identification using ITS sequences generated by nanopore sequencing and compared various methods for the analysis of nanopore sequences. Finally, we developed a new sequence analysis pipeline for species identification using ITS sequences, which facilitates a better interpretation of sequence data obtained by NGS, which has the potential to be applied to metabarcoding data analysis, leading to the identification of sequence reads to the species level.

### Supplementary Information

**Additional file 1: Table S1.** The metadata of the eight yeast strains in this study.

**Additional file 2: Table S2.** The command lines for each of the programs used in this study.

**Additional file 3: Figure S1.** The throughput and number of passes reads per barcode. (**a**) The throughput in terms of base pairs per minute. (**b**) The number of passed reads per barcode.

**Additional file 4: Figure S2.** Information of the three mixed read scenarios.

**Additional file 5: Figure S3.** Classification results of nanopore raw sequence data using WIMP from EPI2ME™ pipeline. The standard classification result displayed from the EPI2ME™ pipeline.

**Additional file 6: Figure S4.** Number of clusters for each clustering method. (**a**) The number of clusters for each clustering method per mix read scenario. (**b**) The number of clusters for each clustering method per barcode.

## Data Availability

The raw sequences from Sanger sequencing were submitted to GenBank with the accession numbers OP404259-OP404266. The raw sequences from nanopore sequencing were submitted to Sequence Read Archive (SRA) database under the BioProject ID PRJNA878646 with SRA accession numbers SRR21494940-SRR21494933. The sequence generating for re-identification with Sanger sequencing was submitted to the GenBank database with the accession of OP404259-OP404266. The executable file ont_clustering.py was generated under the project name ont_clustering. The source code and documentation were freely available at https://gitlab.com/piroonj/ont_clustering under MIT License. The program was written in Python and compatible with Linux and MacOSX. The executable file umap_consensus.sh was generated under the project name umap_consensus The source code and documentation were freely available at https://github.com/Nattapong4321/Umap_consensus under MIT License. The program was written in shell script and compatible with Linux. The datasets used and/or analyzed during the current study are available from the corresponding author upon reasonable request.
