# Supplementary material for: Targeted sequencing analysis pipeline for species identification of human pathogenic fungi using long-read nanopore sequencing
Source: IMA Fungus. 2023 Sep 6;14:18. doi: 10.1186/s43008-023-00125-6 (PMC10483712; doi:10.1186/s43008-023-00125-6)
Supplement: Supplementary file 1 — Additional file 1: Table S1. The metadata of the eight yeast strains in this study. [file 43008_2023_125_MOESM1_ESM.docx]

**Supplementary Table 1**: The metadata of the eight yeast strains

| **Species** | **Strain** | **Barcode** | **Isolated source** | **GenBank accession** | **Identification /confirmation** |  |
| --- | --- | --- | --- | --- | --- | --- |
| *Nakaseomyces glabratus* (formerly *Candida glabrata*) | CUCGL001 | barcode 1 | Sputum | OP404259 | VitekII™/ PCR |  |
|  |  |  |  |  |  |  |
| *Trichosporon asahii* | CUTAS002 | barcode 2 | Nail | OP404260 | VitekII™/ PCR |  |
| *Pichia kudriavzevii* (formerly *Candida krusei*) | CUCKR003 | barcode 3 | Sputum | OP404261 | VitekII™/ PCR |  |
|  |  |  |  |  |  |  |
| *Cryptococcus neoformans* | CUCNE004 | barcode 4 | Hemoculture | OP404262 | VitekII™/ PCR |  |
| *Candida albicans* | CUCAL005 | barcode 5 | Hemoculture | OP404263 | VitekII™/ PCR |  |
| *Candida tropicalis* | CUCTR006 | barcode 6 | Stool | OP404264 | VitekII™/ PCR |  |
| *Trichosporon asahii* | CUTAS007 | barcode 7 | Nail | OP404265 | VitekII™/ PCR |  |
| *Candida albicans* | CUCAL008 | barcode 8 | Sputum | OP404266 | VitekII™/ PCR |  |
