# Supplementary material for: Targeted sequencing analysis pipeline for species identification of human pathogenic fungi using long-read nanopore sequencing
Source: IMA Fungus. 2023 Sep 6;14:18. doi: 10.1186/s43008-023-00125-6 (PMC10483712; doi:10.1186/s43008-023-00125-6)
Supplement: Supplementary file 2 — Additional file 2: Table S2. The command lines for each of the programs used in this study. [file 43008_2023_125_MOESM2_ESM.docx]

**Supplementary Table 2**: The command lines for each of the programs used in the study

| **Software** | **Command** |
| --- | --- |
| Guppy v5.1.16 | ~/programs/ont_guppy/bin/guppy_basecaller -i ./fast5 --save_path ~/Directory_for_saving_basecalled_fastq -c ~/programs/ont_guppy/data/dna_r9.4.1_450bps_sup.cfg --barcode_kits EXP-NBD104 --trim_barcodes --min_qscore 15 -x auto |
| Filtlong | cd Filtlong  bin/filtlong --min_mean_q 20 --mean_q_weight 10 ./.fastq > ./filtered.fastq |
| Canu v2.2 | canu -p name_of_assembly -d ./directory_to_assembly -nanopore-raw ./.fastq genomeSize=1000 minOverlapLength=integer_depend_on_amplicon_length minReadLength= integer_depend_on_amplicon_length useGrid=false readSamplingCoverage=100 contigFilter="2 0 1.0 0.5 0" |
| VSEARCH v2.21.1 | #truncate read  vsearch --fastx_filter ./.fastq --fastq_trunclen 100_length --fastaout ./truncated_filter.fasta --fastq_qmax 90  #sort read by abundance  vsearch --sortbysize ./_truncated_filter.fasta --output ./_truncated_filter_sorted.fasta --minsize 1  #cluster read by abundance  vsearch --cluster_size ./_truncated_filter_sorted.fasta --consout ./otu.fasta --id 0.97 --clusterout_sort  #filter out chimera from the cluster  vsearch --uchime_denovo otus.fasta --nonchimeras otus_checked.fasta --chimeras chimeras.fasta |
| BLAST+ 2.13.0 | ./blastn -db nt -query ./path_to_reference_seq -outfmt 18 -max_target_seqs 1 -out ./blast_species.txt -remote |
| Modified NanoCLUST | python ./ont_cluster.py -t 4 -r /path_to_fastq_file -k 6 |
