# Supplementary figures and images for: Targeted sequencing analysis pipeline for species identification of human pathogenic fungi using long-read nanopore sequencing
Source: IMA Fungus. 2023 Sep 6;14:18. doi: 10.1186/s43008-023-00125-6 (PMC10483712; doi:10.1186/s43008-023-00125-6)

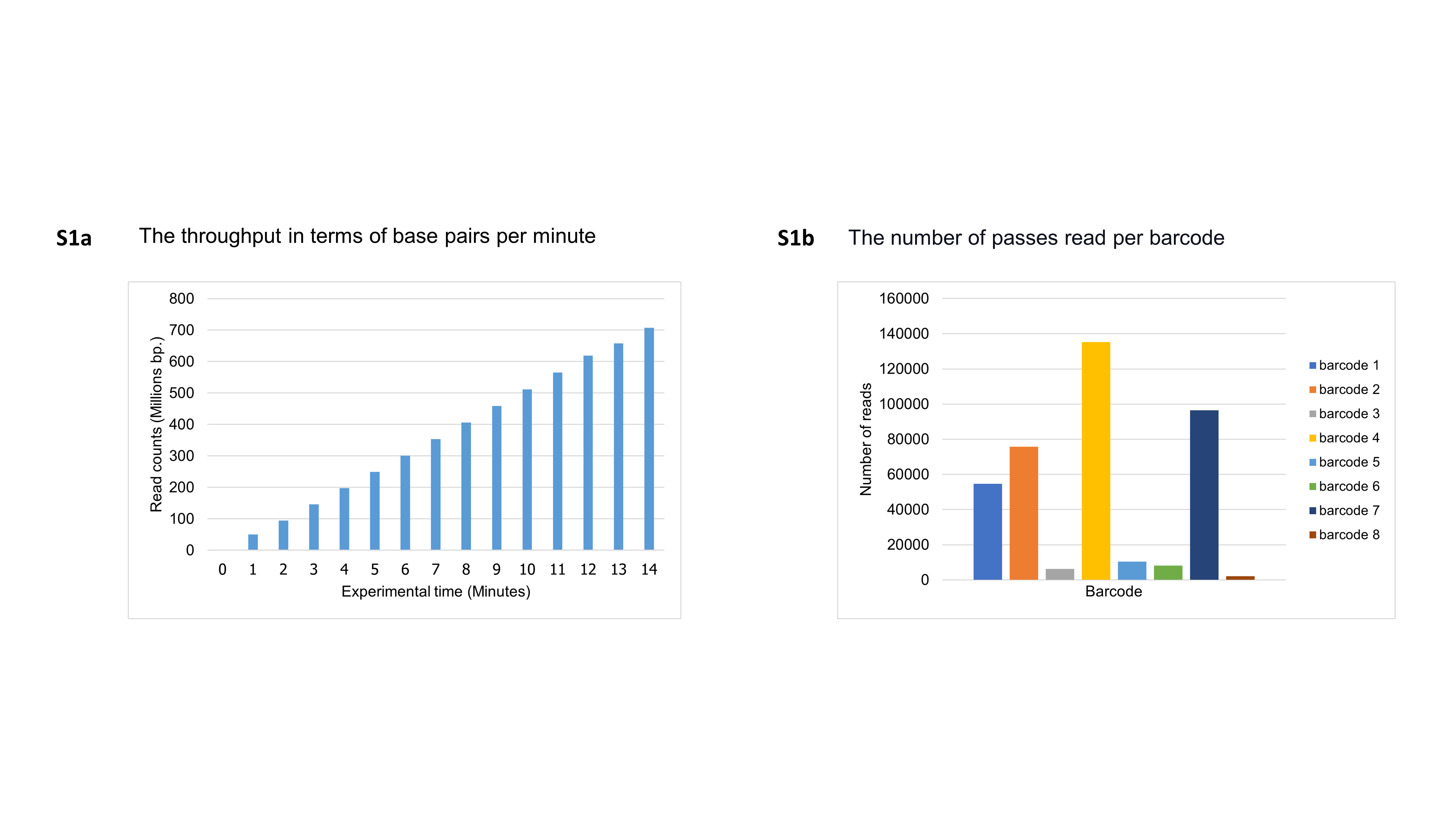

Supplement: Supplementary file 3 — Additional file 3: Figure S1. The throughput and number of passes reads per barcode. (a) The throughput in terms of base pairs per minute. (b) The number of passed reads per barcode. [file 43008_2023_125_MOESM3_ESM.png]

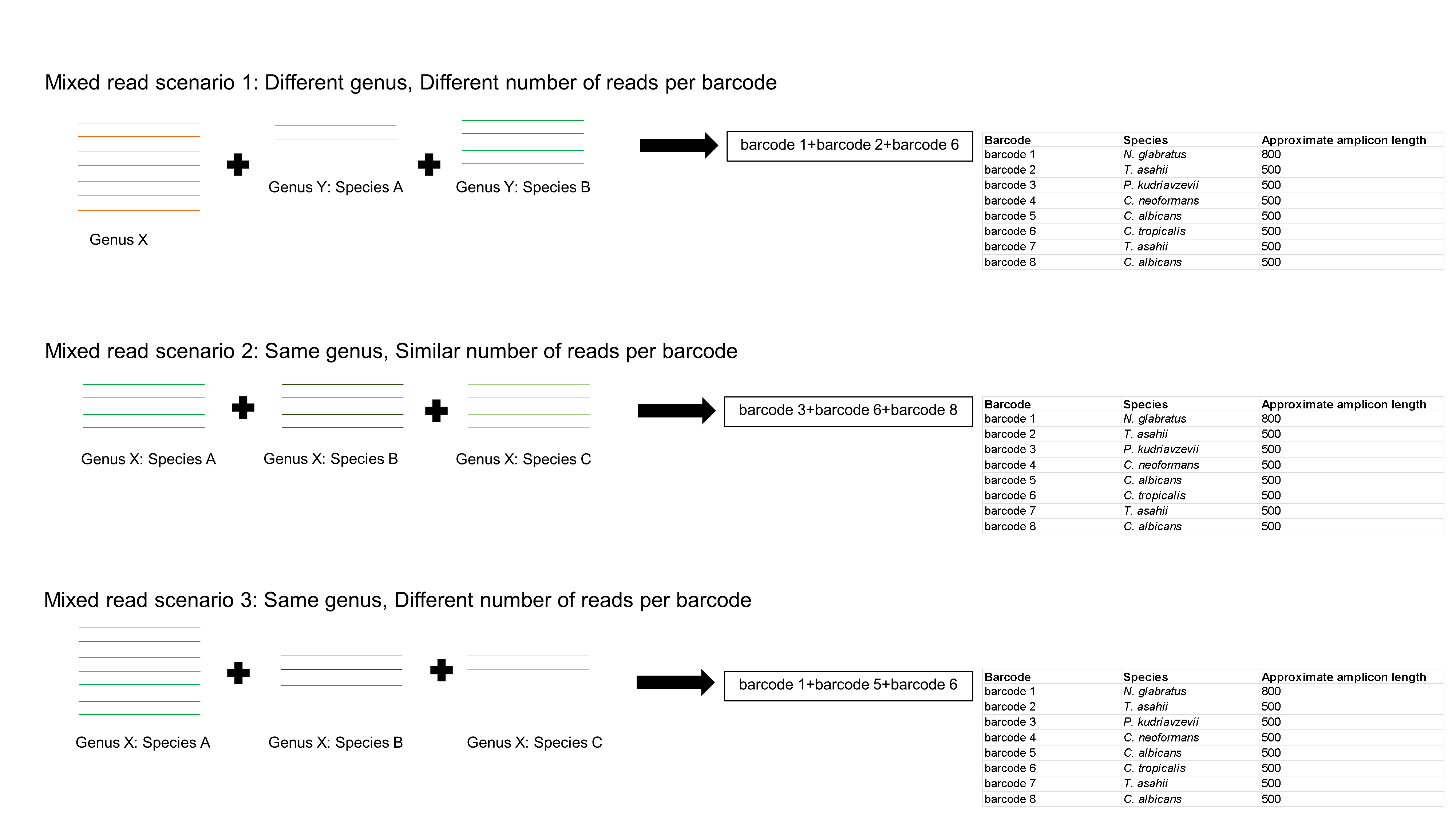

Supplement: Supplementary file 4 — Additional file 4: Figure S2. Information of the three mixed read scenarios. [file 43008_2023_125_MOESM4_ESM.png]

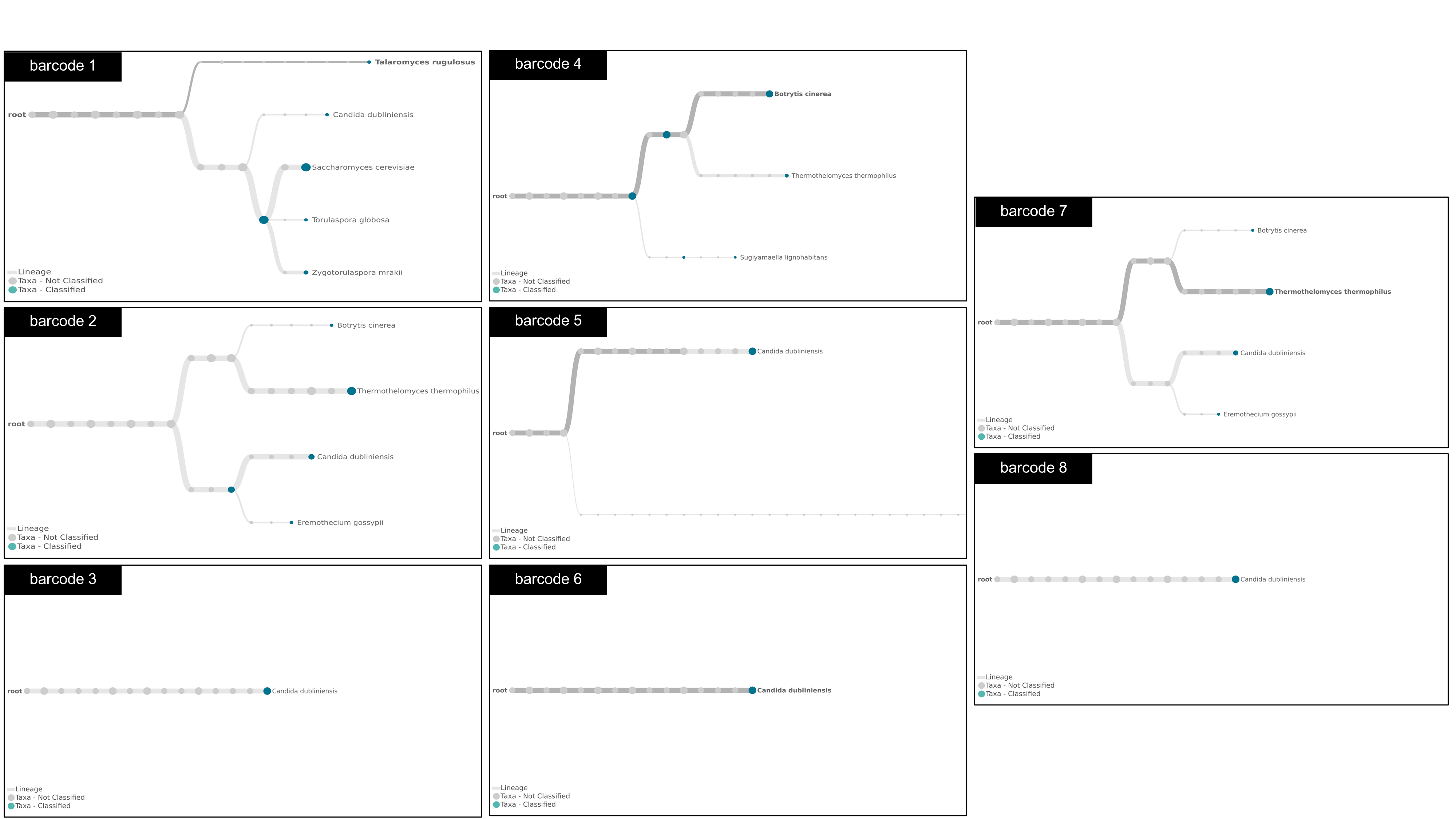

Supplement: Supplementary file 5 — Additional file 5: Figure S3. Classification results of nanopore raw sequence data using WIMP from EPI2ME™ pipeline. The standard classification result displayed from the EPI2ME™ pipeline. [file 43008_2023_125_MOESM5_ESM.png]

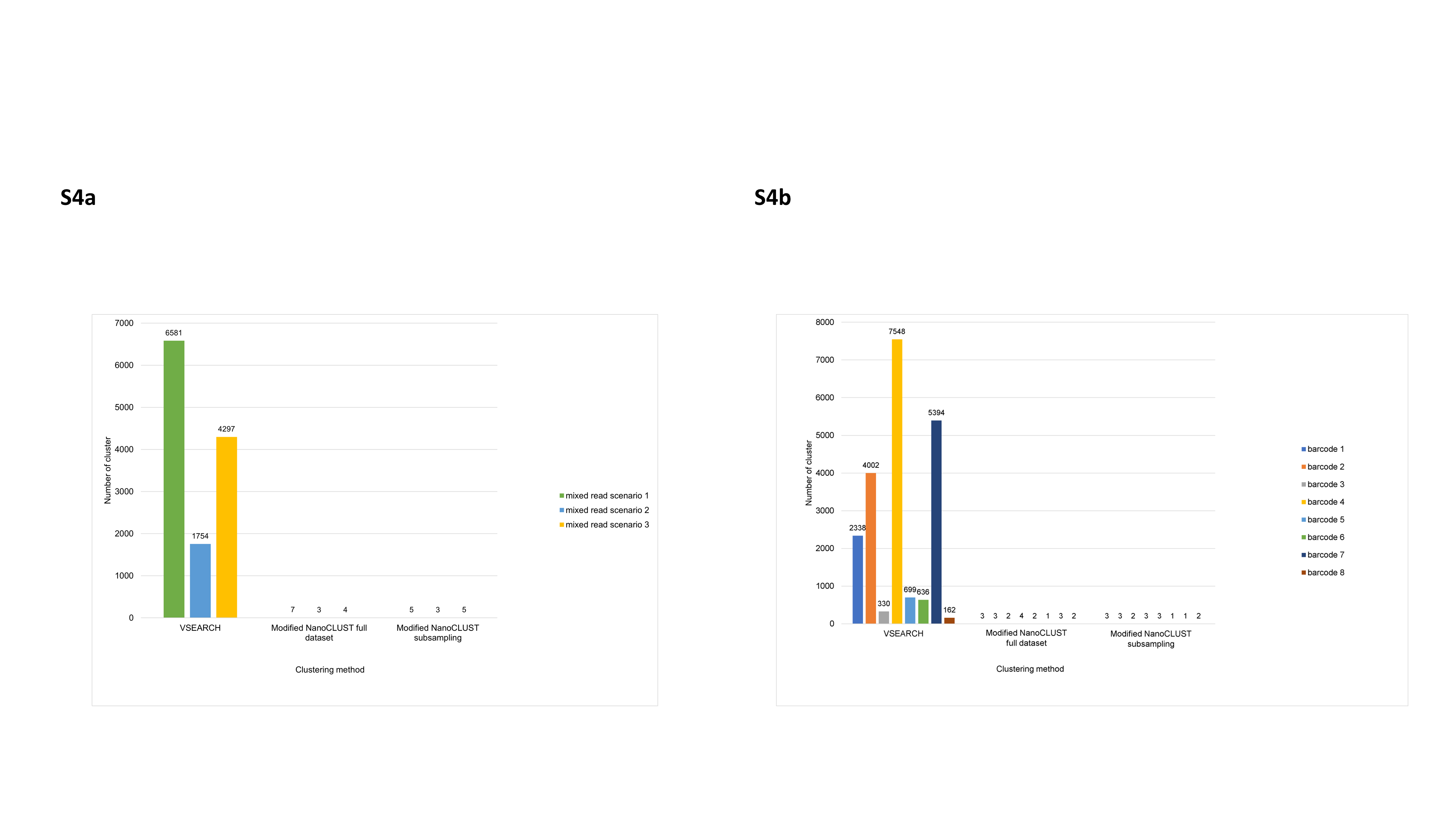

Supplement: Supplementary file 6 — Additional file 6: Figure S4. Number of clusters for each clustering method. (a) The number of clusters for each clustering method per mix read scenario. (b) The number of clusters for each clustering method per barcode. [file 43008_2023_125_MOESM6_ESM.png]
